# Barrier Dispersion-Based Coatings Containing Natural and Paraffin Waxes

**DOI:** 10.3390/molecules27030930

**Published:** 2022-01-29

**Authors:** Julia Woch, Edyta Małachowska, Kamil Korasiak, Aneta Lipkiewicz, Marcin Dubowik, Justyna Chrobak, Jolanta Iłowska, Piotr Przybysz

**Affiliations:** 1 Łukasiewicz Research Network–Institute of Heavy Organic Synthesis “Blachownia”, 47-225 Kędzierzyn-Koźle, Poland; julia.woch@icso.lukasiewicz.gov.pl (J.W.); kamil.korasiak@icso.lukasiewicz.gov.pl (K.K.); justyna.chrobak@icso.lukasiewicz.gov.pl (J.C.); jolanta.ilowska@icso.lukasiewicz.gov.pl (J.I.); 2 Natural Fibers Advanced Technologies, 42A Blekitna Str., 93-322 Lodz, Poland; an_eta12@wp.pl (A.L.); marcin.dubowik1989@gmail.com (M.D.); piotrprzybysz@interia.pl (P.P.); 3 Institute of Wood Sciences and Furniture, Warsaw University of Life Sciences-SGGW, 159 Nowoursynowska Str., 02-787 Warsaw, Poland

**Keywords:** active packaging, dispersion coating, PFAS replacement

## Abstract

Petroleum, synthetic, and natural waxes have been used as hydrophobic bases for dispersions intended for use as barrier coatings for packaging paper. Oil-in-water dispersions with alkaline pH were prepared by a two-step homogenization procedure containing paraffin wax, with various characteristics, the Fischer–Tropsch synthesis product or beeswax. The size of the dispersed particles determined by dynamic light scattering depended on the type of hydrophobic base used and was in the range of 350–440 nm. The ability of dispersion particles in aggregation driven by electrostatic attraction, evaluated by Zeta potential analysis by electrophoretic light scattering, was from −26 to −50 mV. Static multiply light scattering was used for 30 days of stability assessment and helped to select the dispersion with a Sarawax SX70 wax base as the most stable. Dispersions were further used for coating the backing of kraft paper by the Meyer rod method. Coated paper with an applied coating of 6 g/m^2^ had very good hydrophobic properties (Cobb60 < 4 g/m^2^), sufficient strength properties, and air permeation, which enabled its application as a packaging material. The dispersions based on Sarawax SX70 wax were evaluated as the best coating for Mondi ProVantage Kraftliner 125 g/m^2^ backing paper. Good hydrophobic properties and strength properties indicate the possibility of using the SX70-based wax dispersion coating as a replacement for PFAS coatings in some applications.

## 1. Introduction

Paper packaging is a quickly growing (and extremely promising market) for the paper industry. Paper packaging materials made from cellulosic fibers have the advantages of large availability, renewability, biodegradability, and recyclability [1]. Furthermore, paper packaging materials are flexible, inexpensive, and safe [2], and their good printability and functionality make them competitive in the packaging field. Considering the functional properties of paper packaging, one of the major requirements for packaging papers is the need to provide a moisture barrier [3]. However, its porous nature prevents paper from fulfilling the protective function and makes its application in the packaging sector limited, especially in humid conditions, because excessive liquid absorption not only makes the paper structure weaker [4,5], but also leads to poor printing quality [6]. Due to the hydrophilic character of the cellulose fibers arising from the multiple hydroxyl groups, packaging paper grades are hydrophobized to reduce penetration and wetting by water [7,8]. In the paper manufacturing industry, this treatment is frequently performed throughout the coating process, allowing the control of penetration of liquids into the paper [9]. The surface coating of paper depends on a combination of several factors, such as the properties of the coating mixture (chemical composition, viscosity, temperature, pH) and the base paper properties (e.g., basis weight, porosity, surface roughness). The base paper must have the right hydrophobicity, which depends on its intended use. In the paper packaging industry, kraft paper is commonly used because of its low cost of production. Therefore, kraft paper has to be coated with a good moisture barrier material (because of the high porosity and large pore size in such paper [10,11]) that will not create problems in recycling and will have minimal impact on its cost [12,13].

Different types of synthetic and natural waxes provide a relatively good moisture barrier and grease resistance properties. Significantly, these substances are relatively cheap and readily available, with versatile use, starting with coatings [14] to catalysis [15], health science [16] to cultural heritage treatment [17], and other innovative applications [18]. Although wax coating is still common, it has been mostly superseded by plastic films ensuring superior performance [19,20,21,22,23]. However, the use of synthetic polymers caused the loss of biodegradability and recyclability of the paper [24,25,26]. The desire to reduce packaging waste whilst keeping other usable properties created an interest in materials that can replace synthetic films [27,28,29,30,31,32]. It is anticipated that successful coatings will allow greater and more effective use of kraft paper in daily life.

In addition to advances in coating materials, progress has also been made in the technology of the paper coating process, considering the application and distribution of the coating material (from brush-like paints in early processes to the currently used roll or jet applicators). Hydrophobic barrier coatings are most likely to be applied on paper by means of extrusion [33], lamination [34], and dispersion coating [35]. The dispersion methodology is preferred due to the better digestion and compatibility of the aqueous system [36]. The gravure roll coaters are most often used for precise management of the coating thickness using a blade or a metering rod [37,38]. In fact, progress in both the paper coating materials and the coating process stemmed from the advent and needs of the packaging [39,40] as well as printing [41] industries.

Active packaging is a type of packaging that has an impact on extension of the shelf life and the maintenance, or even improvement, of the quality of its content, which is mostly food [42,43,44,45]. Research has shown that paper coated with some active ingredients, such as waxes, proteins, antioxidants, and antimicrobials, may be an efficient active packaging material [46]. In the ACTIPAC project, a dispersion composition intended for coating paper used as material for active packaging, ultimately for storage of certified soybean seeds, is being developed. A wax-based composition has been used to provide a moisture barrier in kraft paper.

In this study, oil-in-water dispersions based on different waxes were obtained and their physicochemical properties were designated. Properties of paraffins and waxes used for this study are shown in Table 1.

The effect of the particular wax base on the resulting barrier, structural, and tensile properties of the coated paper was evaluated to find out the optimum combination of wax bases, which will ensure the most usable properties of papers intended for packaging. Research describes how to select a combination of coating mixture to yield a specified surface hydrophobicity level for paper substrates. The paper with the optimal properties will be used as a material for packaging bags for the storage of soybean seeds, where the paper and the coating are completely biodegradable.

## 2. Results

The following results are presented: the composition and properties of dispersions (Section 2.1) and the properties of the coated paper (Section 2.2).

### 2.1. Dispersions: Composition and Properties

**Figure 1:**
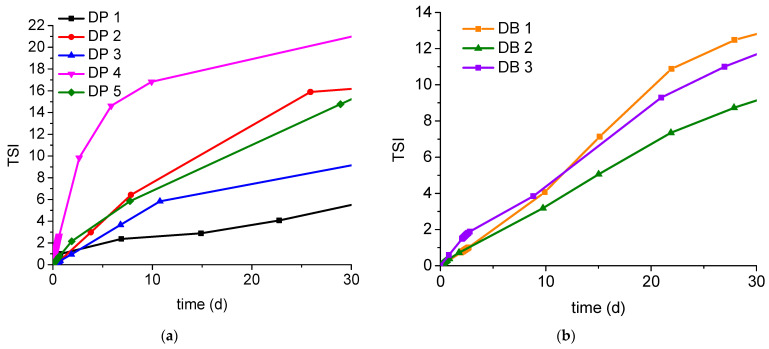
Comparison between Turbiscan Stability Index (TSI) of: (**a**) dispersions containing paraffin (petroleum and synthetic) waxes with constant additive packs (DP series); (**b**) dispersions containing beeswax with different emulsifiers. The lower the TSI value, the less changes inside the sample (DB series).

Two series of dispersions, paraffin based (DP) and beeswax based (DB), were prepared. The first one was used to discover the influence of paraffin base composition on the dispersion properties. To do so, five mixtures of LTP 56/25 paraffin with different synthetic and non-synthetic paraffins and waxes were compared. The second one was essentially used to establish the best emulsifier. The compositions of the obtained dispersions are listed in Table 2. The properties of the obtained dispersions are listed in Table 3. A comparison between each sample’s Turbiscan Stability Index (TSI) is shown in Figure 1.

### 2.2. Properties of the Coated Papers

Sensory evaluation of the samples (the occurrence of piercing) was presented in Table 4. 

Based on 3D microscopic photos of the coated surfaces papers, roughness profiles were prepared (Table 5), i.e., profiles obtained from the surface profile by separating the long-wave components of the profile (waviness and shape deviations) with an λc profile filter. The λc-profile filter determines the transition from roughness to waviness, i.e., random or close to periodic inequalities. Table 5 also summarizes the basic roughness parameters (S_a_ and S_z_) describing the surface microgeometry and relating to specific profile features. For roughness profiles, Sa stands for the arithmetic mean deviation of the roughness profile along the sampling length, while S_z_ stands for the maximum roughness (the maximum height of the profile indicates the absolute vertical distance between the maximum profile peak height and the maximum profile valley depth along the sampling length). 

Properties of the coated samples (air permeability, roughness, hydrophobicity, tensile properties) are listed in Table 6. The strength properties were determined for both the machine and cross directions. However, it should be emphasized at this point that the real practical value represents only the results obtained for the machine direction, because it is in this direction where processing processes are conducted in the so-called web processing machines.

## 3. Discussion

### 3.1. Paraffin Dispersions (DP)

The influence of paraffin/wax phase composition on dispersion and coating parameters was evaluated. Each of the paraffin/wax phase was made from two ingredients. One of the ingredients, paraffin LTP 56/25, was constant for all of the samples, and the second ingredient varied (Table 2). The composition of the emulsifiers and additives was also constant for all of the samples. Dispersion samples were made following the procedure described in p. 3.4.1. All of the samples were milky dispersions with dry matter of approximately 41–42 wt% and pH of approximately 9.2–10 (Table 3). Sample DP 1 (containing Sarawax SX70) was the most viscous (34.0 cP), which sometimes could be a disadvantage because of forcing a reduction of the coating speed. However, DP 1 had the greatest stability during the 30-day storage at 25 °C, which is a great advantage. DP 1 also had the greatest zeta potential value, which means that its particles have the lowest aggregation tendency. Sample DP 5 (containing R-58 paraffin) had the lowest viscosity (21.5 cP). However, DP 5 has poor stability, far worse than DP 1. Samples DP 2–4 have (a bit) greater viscosity values than DP 5, but still significantly lower than DP 1. However, all of them have far worse stability than DP 1 (Table 3, Figure 1). In conclusion, DP 5 is the best sample taking into account the criterion of viscosity, but in terms of stability, DP 1 is the best sample.

As part of the sensory analysis of the tested samples, the occurrence of punctures in the applied coating was assessed. As a coating, DP 1 is the best sample. The analysis of the images juxtaposed in Table 4 shows that only this coating provides no piercing to the other side of the paper. The remainder of the coating mixtures (DP 2–DP 5) penetrated the paper structure too much during the fusing process, which resulted in the phenomenon of piercing on its reverse. The coating pierce phenomenon on the coated paper reverse is an undesirable effect of the coating strengthening process. The presence of punctures on the reverse side can cause many problems during further processing of paper (especially during gluing and printing). 

When processing paper, the roughness of the material’s surface is also a very important parameter because it conditions many properties of the coated material (e.g., the thickness of the coating when coating a material). Differences in surface roughness also affect a variety of other characteristics, such as appearance and aesthetics of the product. Smooth surfaces with low asperity tend to have shiny or even mirror-like reflections, while rough surfaces with high asperity will diffusely reflect light. Roughness of the sample-coated DP 1 mixture, measured by the Bendtsen method, is substantially lower from other samples, but it depends on the application if it is a disadvantage or not. For the remaining samples (DP 2–DP 5), no significant differences were found (Table 6). The microscopic analysis of the roughness also showed no significant differences in this group of coating mixtures (Table 5), which is in line with the results of other researchers, and also comparable to other types of coatings [48].

As known, the tensile properties of paper sheets are of great importance for the manufacturing and printing process [49,50]. Therefore, the mechanical properties of coated papers were tested to evaluate the effect of coating with synthetic and natural waxes. Interestingly, the obtained results of strength are close to the properties of papers with PVA/AKD coatings [27]. Concerning the strength properties, paper coating the DP 1 paraffin dispersion achieved the best results in conducted tests. Coating DP 1 also has the greatest hydrophobicity (Table 3 and Table 6). Good tear resistance, elasticity, and hydrophobicity make the DP 1 dispersion the most attractive for use in the coating process of packaging papers. In addition, it is noted that strength properties of coated papers have better strength in machine direction conditions (which are crucial for the coating process) than in cross direction tests (Table 6).

### 3.2. Beeswax Dispersions (DB)

The influence of the composition of the emulsifiers used in the preparation of dispersions and the coating parameters were evaluated. Wax and glycerol monostearate quantities were constant for all of the samples (Table 2). All of the samples were milky, slightly yellow dispersions, with a dry matter of approximately 32.5 wt% and pH of approximately 8.5–8.8. Generally, all of the samples had low viscosity (approximately 9 cP, Table 3), which make them applicable in various coating techniques. Additionally, there is no substantial influence of the type of used emulsifier on the mean particle size of the dispersion. DB 2, containing alkyl polyglucoside as an emulsifier, had the greatest stability. However, all three samples are characterized by similar values of zeta potential (Table 3), which means that the destabilization mechanisms probably do not depend on the aggregation.

Papers coated with DB 2 and DB 3 samples have the best strength properties; however, DB 2 provides significantly better properties in the cross direction than DB 3 (Table 6). In turn, beeswax dispersions, especially DB 1 and DB 2, increase the barrier properties of paper and simultaneously decrease the liquid permeation to a greater extent than DP mixtures (Table 5), which coincides with the previous considerations in the available literature, as well as for other types of coatings [27,51]. Each coating based on beeswax can ultimately be useful for a variety of applications (including anti-bioadhesion), specifically where light-weight, reasonably priced, environment-friendly or disposable hydrophobic products are desired [52,53,54]. Therefore, each beeswax dispersion, depending on the intended use of the packaging paper, can potentially be used in converting processes.

Interestingly, the strength properties of papers coated of paraffin and beeswax dispersions are on a similar level, despite significant differences in viscosity of the coating mixtures. This is a very interesting observation compared to the results obtained by other researchers [55]. In theory, with highly viscous mixtures, it is harder to form a uniform coating, while for mixtures with low viscosity, it is easier to get a flat and uniform coating, leading to a better warp-resistance and increased strength of the paper sheet after the coating. In addition, as a result of lower viscosity of the coating mixture, the more uniform coating and compact surface of DB-coated papers are beneficial toward improving the other barrier properties of end products, such as air permeability (Table 6), which is good for the final application in high quality packaging [56]. Furthermore, the coating layer’s porosity and the surface roughness affect the interaction between printing inks and the paper. Thus, how well a coated paper sheet will reproduce the images when it is printed depends on the uniformity and the coated surface properties [57,58].

All of DB samples provide advantageous non-piercings during their use as coatings (Table 4). Moreover, all of the samples, according to the Bendtsen procedure, provide similar roughness of the coated paper (Table 6), which may arise from the same coating weight and process parameters. The smoothness of coated papers largely depends on the degree of coating uniformity [59]. However, despite the slight differences in roughness measured by the Bendtsen method, significant differences in the roughness values of beeswax dispersion coated samples are visible by using the microscopy technique (Table 5). Moreover, the measurement results obtained by the microscopic technique for the group of samples coated with paraffin dispersions differed significantly from the results obtained for the group of beeswax dispersions. These variations most probably resulted from a large difference in viscosity of individual groups of dispersions. Therefore, it can be concluded that the analysis of linear roughness performed with the microscopic technique allows one to observe changes in surface properties that cannot be observed during the Bendtsen method. 

## 4. Materials and Methods

### 4.1. Materials

Commercially available Mondi ProVantage Kraftliner paper with a basis weight of 125 g/m² was used for the coating process. 

Commercially available synthetic and natural waxes were used as the hydrophobic bases for the dispersions. A mixture of paraffin waxes containing C10–C40 hydrocarbons, Paraffin LTP (technical grade), CAS number: 8002-74-2, was purchased from Polwax, Jasło, Poland. The Fischer–Tropsch synthesis product—Shell Sarawax SX70 (technical grade), CAS 8002-74-2 was obtained from Shell Deutschland Oil GmbH, Hamburg, Germany. K60 wax, K70 wax, HT wax (technical grade, each of them a mixture of petroleum wax with no CAS specified), and Paraffin R-58 (technical grade, refined by bleaching earth, CAS 6742-43-4) were obtained from ORLEN Południe, Trzebinia, Poland. Natural yellow beeswax (technical grade, for cosmetics purposes) was manufactured in China. Stearic acid (pure) was obtained from Centro-Chem, Lublin, Poland. Glycerol monostearate (technical grade) was obtained from Louis François, Croissy-Beaubourg, France. Glucoside-based emulsifiers (technical grade) were obtained from Colonial Chemical, Inc., South Pittsburg, USA. Sodium hydroxide (pure p.a.) was obtained from Stanlab, Lublin, Poland. Potassium hydroxide (pure p.a.) was obtained from Stanlab, Lublin, Poland. The water used for the preparation of the dispersions was a distilled water.

### 4.2. Preparation of Coating Dispersions

Approximately 1 kg of a sample was prepared via a two-step homogenization procedure. Quantities of paraffins and waxes (by %wt) are shown in Table 2. At the first step, a pre-emulsion mixture was prepared: the wax phase-containing wax, emulsifiers, and oil-soluble additives were heated up to 95 °C in a glass reactor equipped with a thermometer and reflux condenser and was stirred using a mechanical stirrer for 20 min. The water phase, which was a NaOH or KOH solution (details shown in Table 1), was prepared separately in a glass beaker and heated up to 95 °C and was added partially to the homogenous wax phase. The hydroxides were used to saponify fatty acids in the oil phase. The mixture was stirred to obtain a low-viscous milky pre-emulsion for 15 min. Then, the obtained pre-emulsion was moved into the pressure homogenizer (PANDA Plus) with a two-step homogenization system. The homogenization procedure was repeated twice for each sample.

### 4.3. Dynamic Viscosity

Dynamic viscosity was determined using a Brookfield RVDV-II+ viscometer. The measurements were carried out at 23 °C, using a spindle no. 28, at 100 rpm.

### 4.4. Basic Physicochemical Parameters

pH was determined using a Schott Lab 850 pH meter. Dry matter was determined as the difference in the mass of dispersion samples before and after 2 h of heating in laboratory heater at 120 ± 2 °C.

### 4.5. Evaluation of the Stability of the Dispersion in Static Conditions

The stability of the dispersions was evaluated by measuring the intensity of the backward reflected light (BS) as a function of the height of the sample during 30 days of storage at 25 °C. Measurements were performed at 25 °C using a Turbiscan Thermo (Formulaction, France) equipped with a light source with a wavelength of 880 nm. The detector registered a backscattered light signal (BS).

(1) $$BS=\frac{1}{\sqrt{l^{*}}}\;\mathrm{and}\;l^{*}\left(\varphi ,d\right)=\frac{2d}{3\varphi \left(1-g\right)Q_{s}},\;$$

where $l^{*}$ is the average path of a photon inside the dispersion, φ is a volume fraction of particles, d is the mean particle diameter, and g and $Q_{s}$ are the optical parameters obtained by Mie light scattering theory [60]. The variation in *BS* corresponds to the changes in the microstructure of the dispersions, such as particle migration or changes in their size.

The undiluted dispersion samples were studied in cylindrical glass vials. Before the measurements, every sample was hand mixed and left for degassing for at least 2 h.

For qualitative evaluation of the observed changes in the sample, the Turbiscan Stability Index (TSI) was used. TSI was calculated using TurbiSoft software and Formula (2), based on all data obtained by static multiply light scattering (SMLS). A higher TSI corresponds to more changes occurring in the studied sample.

(2) $$TSI=\sqrt{\frac{\sum_{i=1}^{n}\;{\left(x_{i}-x_{BS}\right)}^{2}}{n-1}}$$

where *x_i_* (from *i* = 1 to *n*) is the mean value of backscattering, *x_BS_* is the mean value of *x_i_*, and *n* is the number

### 4.6. Dispersion Droplet Size Determination

Dynamic light scattering (DLS) was used to determine the average size and size distribution of the dispersion particles. The measurements were performed with a Zetasizer Nano ZS (Malvern Panalytical Ltd, Malvern, United Kingdom) with a He–Ne laser beam of wavelength λ = 633 nm. All measurements were carried out at an angle of 173° at 25 °C. The autocorrelation functions were analyzed by the CONTIN algorithm. The dispersity of particle sizes was given as $\frac{\mu_{2}}{{\overset{¯}{\Gamma }}^{2}}$, where Γ is the average relaxation rate and *µ*_2_ is its second moment; both values were obtained from cumulant analysis. The apparent hydrodynamic diameter of the dispersion particles was calculated using the Stokes–Einstein Equation (3).

(3) $$D=\frac{kT}{3\pi \eta D_{H}}$$

where *D* is the translational diffusion coefficient (m^2^·s^−1^), *k* is the Boltzmann’s constant, *T* is the temperature (K), *η* is the dynamic viscosity of dispersant (Pa·s), *D_H_* is the hydrodynamic diameter (m).

### 4.7. Zeta Potential of the Dispersions

Zeta potential (ZP) was measured using a Zetasizer Nano ZS (Malvern Panalytical Ltd., Malvern, UK) with a He–Ne laser beam of wavelength λ = 633 nm. The electrophoretic light scattering data were collected at an angle of 13°. The Smoluchowski model was used to calculate Zeta potential values. Zeta potential measurements were performed for 1 vol% dispersions, prepared with double distilled and filtered water, as used for the DLS measurements.

### 4.8. Coating Process of the Paper

For all tests, the commercially available Mondi ProVantage Kraftliner paper with a base weight of 125 g/m² was used.

**Figure 2:**
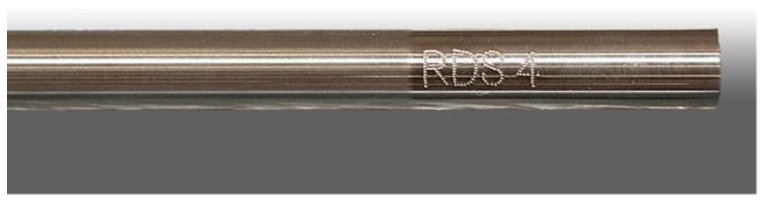
Sample photo of Mayer rod.

Before the coating process, paper samples were cut from the rolls and subjected to air conditioning at 23 °C and 50% relative humidity, according to ISO 187:1990, for a minimum of 24 h. The coating process was carried out with the use of Mayer rods based on the TAPPI T 552 standard. A photo of one of the rods used is shown in Figure 2. For each of the coating mixtures, at least 20 coating operations were performed, which differed, among other factors, from the dynamics of guiding the coating rod. The use of different coating rod guidance dynamics was aimed at the preliminary elimination of a coating showing shear thickening behavior, which causes technological difficulties when working in a continuous operation system. After the coating process, the coated paper samples were dried in a thermal research chamber (WAMED, model KBC-65W) at a temperature of 60 °C for 5 min. The deliberate procedure was to set a relatively long drying time. The predetermined drying length of the coated paper sample guaranteed the web dryness effect. This effect reflected the extremely inadequate conditions, which, despite the lack of recommendations, may occur during the production process.

### 4.9. Paper Properties Analysis

The coated paper samples were conditioned at 23 °C and 50% relative humidity according to ISO 187:1990 for a minimum of 24 h before the examination was conducted.

Sensory evaluation of the rheological stability of the coating mixture was performed during its spreading on the surface of the paper. Sensory evaluation of the obtained coating after the paper drying process was performed, and the uniformity of the formed coating was evaluated by searching for the presence of uncoated places and detection of the coating piercing process on the reverse side of the coated paper. Air permeability was determined according to ISO 5636-3:2013—TMI 58-27 Bendtsen Roughness Tester (Kontech, Lodz, Poland); roughness of the coated paper surface was determined in accordance with ISO 8791-2:2013—TMI 58-27 Bendtsen Roughness Tester (Kontech, Lodz, Poland); microscopic analysis of the paper linear roughness (S_a_ and S_z_) was conducted using the instrumental 3D optical microscopy technique (ISO 25178: 2016)—KEYENCE VHX 7000 equipped with the VH-Z20UT objective; the degree of hydrophobicity and/or hydrophilicity of paper was determined according to ISO 535:2014 (water absorption by Cobb_60_ method) with Cobb apparatus (Danex, Katowice, Poland); and the priority strength properties of base papers, in accordance with ISO 1924-2:2010—Zwick 005 ProLine testing machine (ZwickRoell, Ulm, Germany), were coupled with testXpert III software. The tensile paper properties were examined as follows:

- Breaking length (m);
- Width related force with break (N/m);
- Force at break index (Nm/g);
- Strain at break (%);
- Energy absorption (J/m^2^);
- Energy absorption index (J/g);
- Young’s modulus (Mpa).

A detailed statistical analysis was performed for the individual research series, determining the basic indicators—arithmetic mean, extended deviation, and percentage relative error.

## 5. Conclusions

Synthetic and natural wax coatings were prepared and deposited onto paper. The results showed that, despite differences in mixture composites and properties, significant differences in tensile properties and roughness of the examined samples were not observed. However, beeswax-based dispersions as coating agents could significantly improve the barrier properties of paper sheets (hydrophobicity almost double; air permeability thrice) compared with paraffin dispersions, possibly because of lower viscosity. Based on the results, we conclude that the viscosity of coating agents must be taken into account, as well as their affinity toward paper within coating formulation and rheology.

## Figures and Tables

**Table 1:** Selected properties of paraffins and waxes used for this study.

Parameter	LTP 56/25 Paraffin	R-58 Paraffin	Sarawax SX70	K60 Wax	K70 Wax	HT Wax	Beeswax
Congealing point, °C	n.s.*	n.s.	68–72	60	n.s.	60–70	n.s.
Freezing point, °C	54–58	54–60	n.s.	n.s.	64–70	n.s.	n.s.
Melting point, °C	n.s.	n.s.	n.s.	n.s.	n.s.	n.s.	approximately 65 °C
Needle penetration at 25 °C, 0.1 mm	max. 25	20	9–16	22–28	10–16	n.s.	n.s.
Oil content, %wt	max. 1.1	max. 0.8	max. 0.5	n.s.	max. 1.0	n.s.	n.s.
Kinematic viscosity at 100 °C, cSt	n.s.	not specified	5–8	5–7	5–7	not specified	8.9 [47]

* n.s.—not specified.

**Table 2:** Composition of obtained dispersions. Quantity by wt%. DP—paraffin-based dispersions, DB—beeswax-based dispersions.

Ingredient	DP 1	DP 2	DP 3	DP 4	DP 5	DB 1	DB 2	DB 3
Beeswax	-	-	-	-	-	30.0	30.0	30.0
LTP 56/25 paraffin	17.0	17.0	17.0	17.2	17.0	-	-	-
R-58 paraffin	-	-	-	-	17.0	-	-	-
Sarawax SX70	17.0	-	-	-	-	-	-	-
K60 wax	-	-	17.0	-	-	-	-	-
K70 wax	-	17.0	-	-	-	-	-	-
HT wax	-	-	-	17.0	-	-	-	-
Hydroxides								
Sodium hydroxide	yes (constant quantity)					No		
Potassium hydroxide	no					yes (constant quantity)		
Additives								
Glycerol monostearate	yes (constant quantity)					yes (constant quantity)		
Stearic acid	yes (constant quantity)					No		
Emulsifier *	1	1	1	1	1	1	2	3

* 1—decylglucosides, 2—alkyl polyglucoside, 3—decylglucoside crosspolymer.

**Table 3:** Properties of obtained dispersions. DP—paraffin-based dispersions, DB—beeswax-based dispersions.

Parameter	DP 1	DP 2	DP 3	DP 4	DP 5	DB 1	DB 2	DB 3
Dynamic viscosity, cP	34.0	24.5	25.5	24.5	21.5	9.5	9.0	8.5
pH	9.96	9.20	9.70	9.70	9.80	8.58	8.71	8.79
Dry matter, wt%	41.8	40.8	41.9	41.0	41.8	32.5	32.5	32.8
Stability, TSI 30 days *	5.5	16.1	9.1	21.0	15.2	12.9	9.1	11.7
Mean particle size, nm	437	401	422	359	358	358	363	375
Polydispersity index	0.260	0.257	0.264	0.245	0.231	0.274	0.252	0.288
Zeta potential, mV	−42.4	−29.8	−34.3	−26.0	−28.4	−49.5	−47.1	−47.6

* See on Figure 1.

**Table 4:** Images of the surface and reverse side of the coated paper. DP—papers coated with paraffin-based dispersions, DB—papers coated with beeswax-based dispersions.

Sample	Front of Coated Sample	Reverse of Coated Sample
DP 1	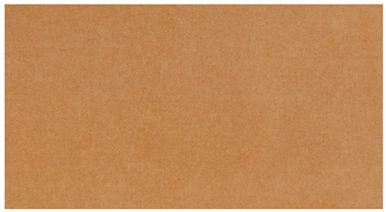	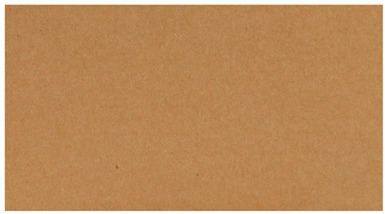
DP 2	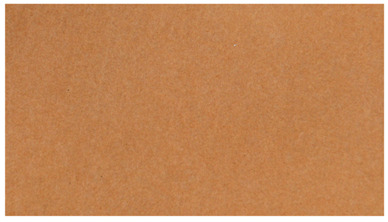	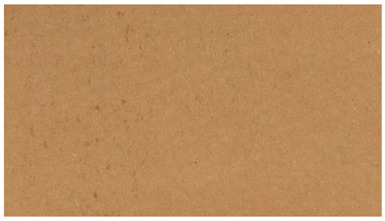
DP 3	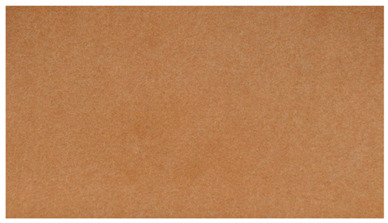	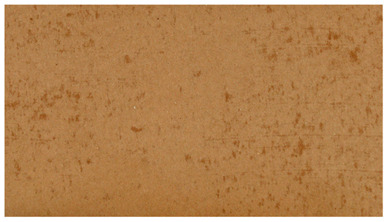
DP 4	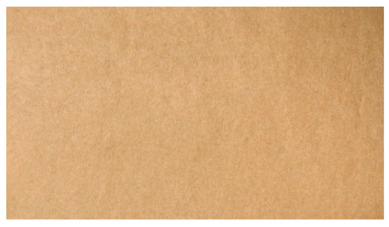	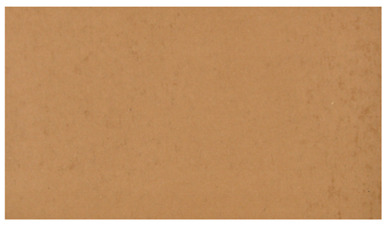
DP 5	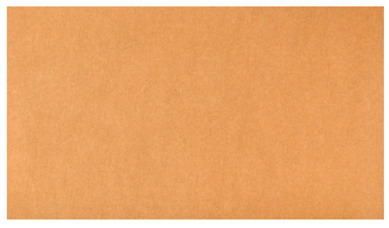	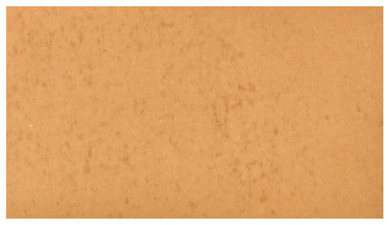
DB 1	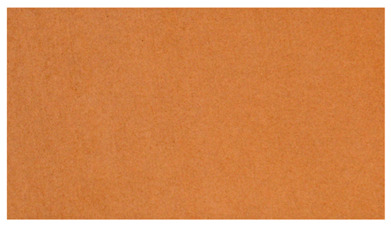	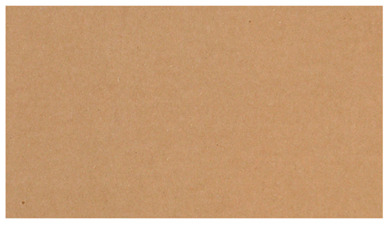
DB 2	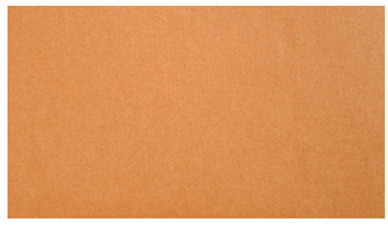	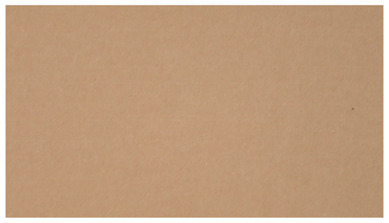
DB 3	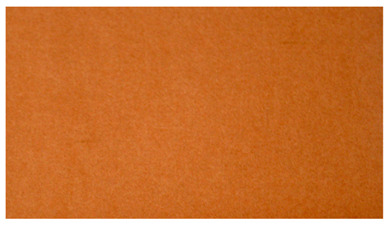	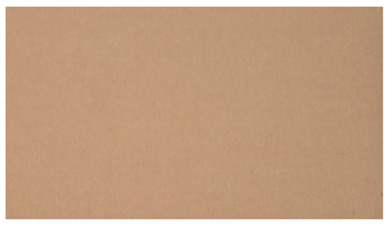

**Table 5:** Roughness profiles of the coated surface of the paper. DP—papers coated with paraffin-based dispersions, DB—papers coated with beeswax-based dispersions.

Sample	Images of Roughness Profiles	S_a_, S_z_ Parameters, µm
DP 1	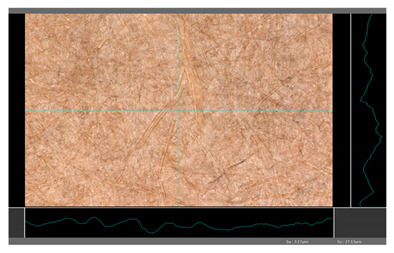	S_a_ = 3.17 S_z_ = 27.13
DP 2	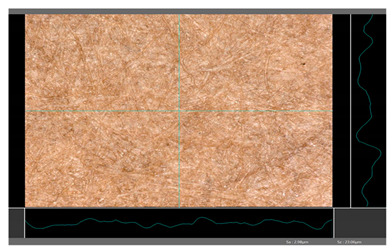	S_a_ = 2.98 S_z_ = 23.06
DP 3	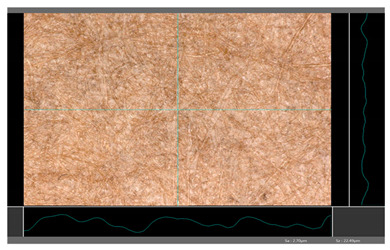	S_a_ = 2.70 S_z_ = 22.49
DP 4	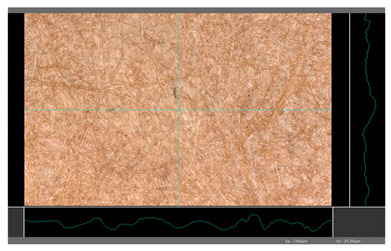	S_a_ = 2.60 S_z_ = 25.10
DP 5	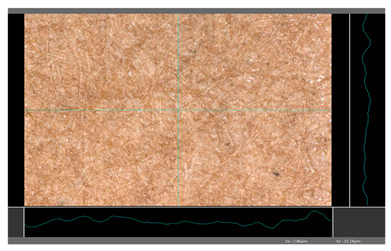	S_a_ = 2.86 S_z_ = 31.18
DB 1	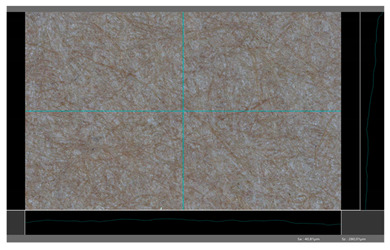	S_a_ = 40.81 S_z_ = 280.01
DB 2	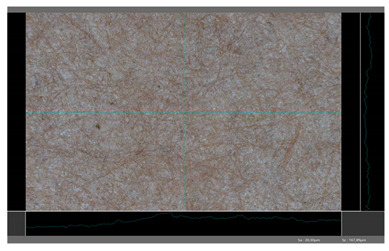	S_a_ = 20.30 S_z_ = 167.89
DB 3	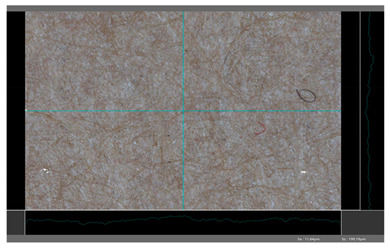	S_a_ = 11.64 S_z_ = 199.19

**Table 6:** Sensory evaluation of the dispersion samples (before the coating process) and properties of the coated papers.

Parameter	DP 1	DP 2	DP 3	DP 4	DP 5	Mean Value	DB 1	DB 2	DB 3	Mean Value
Piercing	no	yes	yes	yes	yes	-	No	no	no	-
Air permeability, mL/min	106	89	98	110	108	102.2	47	29	28	34.7
Roughness, mL/min	320	457	427	387	456	409.4	387	390	377	385
Cobb_60_, g/m^2^	3.90	8.03	7.48	8.10	7.65	7.03	2.98	3.83	5.59	4.13
Strength properties—machine direction										
Breaking length, km	12.32	11.54	11.47	11.93	11.07	11.67	11.78	12.14	11.89	11.94
Tear in break, N	159.1	148.9	150.8	152.4	146.0	151.4	147.5	153.0	152.1	150.9
Width related force with break, N/m	16,020	15,020	15,140	15,410	14,670	15,252	15,050	15,540	15,410	15,333
Force at break index, Nm/g	120.8	113.2	112.4	117.1	108.5	114.4	115.4	119.0	116.5	117.0
Strain at break, %	2.67	2.49	2.58	2.68	2.68	2.62	2.72	2.66	2.77	2.72
Energy absorption, J/m^2^	261.9	226.1	236.0	254.6	235.4	242.8	252.2	257.4	264.6	258.1
Energy absorption index, J/g	1.98	1.70	1.75	1.93	1.74	1.82	1.94	1.97	2.00	1.97
Young’s Modulus, Mpa	11,170	10,890	10,630	10,870	10,261	10,764	10,410	11,030	10,660	10,700
Strength properties—cross direction										
Breaking length, km	2.88	2.59	2.71	2.64	2.63	2.69	2.69	2.92	2.86	2.82
Tear in break, N	37.44	33.43	34.39	33.28	33.43	34.39	34.61	36.90	36.56	36.02
Width related force with break, N/m	3749	3373	3569	3470	3494	3531	3451	3733	3704	3629
Force at break index, Nm/g	28.29	25.37	26.54	25.93	25.85	26.40	26.44	25.58	28.04	26.69
Strain at break, %	4.47	5.13	4.97	5.67	5.54	5.16	5.69	5.77	5.24	5.57
Energy absorption, J/m^2^	119.6	128.9	130.6	141.5	142.4	132.6	145.0	152.9	139.4	145.8
Energy absorption index, J/g	0.903	0.970	0.973	1.073	1.053	0.994	1.109	1.172	1.053	1.111
Young’s Modulus, Mpa	3400	3099	3274	3171	3188	3226	3190	3221	3173	3195

A green color means a more advantageous value than the mean of the series. A red color means the less advantageous, respectively. DP—papers coated with paraffin-based dispersions, DB—papers coated with beeswax-based dispersions.

## Data Availability

The data presented in this study are available upon request from the corresponding author.
